# Study protocol: Audit and Best Practice for Chronic Disease Extension (ABCDE) Project

**DOI:** 10.1186/1472-6963-8-184

**Published:** 2008-09-17

**Authors:** Ross Bailie, Damin Si, Christine Connors, Tarun Weeramanthri, Louise Clark, Michelle Dowden, Lynette O'Donohue, John Condon, Sandra Thompson, Nikki Clelland, Tricia Nagel, Karen Gardner, Alex Brown

**Affiliations:** 1 Menzies School of Health Research, Institute of Advanced Studies, Charles Darwin University, Darwin, Australia; 2 Northern Territory Department of Health and Community Services, Darwin, Australia; 3 Western Australia Department of Health, Perth, Australia; 4 Curtin University, Perth, Australia; 5 Aboriginal Health Council of Western Australia, Perth, Australia; 6 Australian Primary Health Care Research Institute, Australian National University, Canberra, Australia; 7 Centre for Indigenous Vascular and Diabetes Research, Baker Heart Research Institute, Alice Springs, Australia

## Abstract

**Background:**

A growing body of international literature points to the importance of a system approach to improve the quality of care in primary health care settings. Continuous Quality Improvement (CQI) concepts and techniques provide a theoretically coherent and practical way for primary care organisations to identify, address, and overcome the barriers to improvements. The Audit and Best Practice for Chronic Disease (ABCD) study, a CQI-based quality improvement project conducted in Australia's Northern Territory, has demonstrated significant improvements in primary care service systems, in the quality of clinical service delivery and in patient outcomes related to chronic illness care. The aims of the extension phase of this study are to examine factors that influence uptake and sustainability of this type of CQI activity in a variety of Indigenous primary health care organisations in Australia, and to assess the impact of collaborative CQI approaches on prevention and management of chronic illness and health outcomes in Indigenous communities.

**Methods/design:**

The study will be conducted in 40–50 Indigenous community health centres from 4 States/Territories (Northern Territory, Western Australia, New South Wales and Queensland) over a five year period. The project will adopt a participatory, quality improvement approach that features annual cycles of: 1) organisational system assessment and audits of clinical records; 2) feedback to and interpretation of results with participating health centre staff; 3) action planning and goal setting by health centre staff to achieve system changes; and 4) implementation of strategies for change. System assessment will be carried out using a System Assessment Tool and in-depth interviews of key informants. Clinical audit tools include two essential tools that focus on diabetes care audit and preventive service audit, and several optional tools focusing on audits of hypertension, heart disease, renal disease, primary mental health care and health promotion.

The project will be carried out in a form of collaborative characterised by a sequence of annual learning cycles with action periods for CQI activities between each learning cycle.

Key outcome measures include uptake and integration of CQI activities into routine service activity, state of system development, delivery of evidence-based services, intermediate patient outcomes (e.g. blood pressure and glucose control), and health outcomes (complications, hospitalisations and mortality).

**Conclusion:**

The ABCD Extension project will contribute directly to the evidence base on effectiveness of collaborative CQI approaches on prevention and management of chronic disease in Australia's Indigenous communities, and to inform the operational and policy environments that are required to incorporate CQI activities into routine practice.

## Background

A substantial body of international literature and a growing body of recent local literature point to the importance of a systems approach in health service management and quality assurance. Within this literature is evidence that identifies the appropriate organisation of the health centre environment as the key to achieving consistent delivery of health services [1-4]. The health centre environment is recognised as both the main source of quality-of-care problems and as providing the best potential to improve the delivery of preventive services [5].

The international experience working within a Continuous Quality Improvement (CQI) theoretical framework, focused on a cycle of "plan-do-study-act", has contributed significantly to modern system developments [6,7]. Fundamental principles of the CQI process include an emphasis on raising the general level of care rather than focusing on pockets of poor practice, an emphasis on organisation of health care and an approach to understanding and revising processes based on data about the processes themselves[8]. CQI concepts and techniques provide a theoretically coherent and practical way for clinics to organise themselves to identify, address, and overcome the barriers to innovation [9]. A system dropped in from the outside is not likely to fit or become sufficiently tailored and internalised to function well or to be sustained [10]. Four factors that appear to be important in implementing a CQI process in a clinic setting are: awareness, momentum, ownership, and communication [11]. The CQI approach seems likely to build systemic improvements in a way that will ensure the ownership and individualised fit that are necessary to maintain change.

The second category of literature deals with specific interventions within existing systems. For example, systematic reviews have examined interventions for influencing clinician behaviour. One such review of the gap between research and practice summarised interventions that had attempted to promote the implementation of research findings [12]. The most effective interventions were educational outreach visits, reminders alone, or some combination of at least two of: audit and feedback, reminders, local consensus processes and marketing. Distribution of recommendations for clinical care alone was among the least effective interventions.

However, most of the international literature about preventive services in primary health care has been written within the context of developed nations, and particularly from a North American perspective. In recent years literature has examined preventive services in primary health care for indigenous or marginalised populations [4,13], in which the burden of chronic disease is often higher, and the need for chronic disease and preventive care services greater, than in the general populations of which they are a part [4]. In these population groups, primary health care services are often more fragmented as a result of chronic under-funding, staff shortages, and ineffectual organisational systems.

The importance of preventable chronic disease as a factor in the overall poor health status among Australian Indigenous people is well recognised. However, until the Coordinated Care Trials (CCTs) [4,14], there had been little research of preventive health system processes in the remote Indigenous context. The CCTs were characterised by establishment of Aboriginal health boards with purchasing authority and implementation of best practice clinical guidelines supported by purpose-built electronic information systems [14]. The local evaluations of the CCTs provided preliminary evidence of the effectiveness of the 'coordinated care system' [13,15]. However, key challenges for the sustainability of improved patient outcomes included:

▪ Clarification of staff roles in chronic illness care

▪ Firm linking of care plan protocols to work practices

▪ Ensuring appropriate responses to abnormal findings

▪ Robust and ongoing management support for chronic illness care

▪ Promotion of the clients' understanding of their roles in self-management.

The recent development of primary care 'collaboratives' in Australia  and overseas [16,17] have been designed to create structures in which interested organisations can easily learn from each other and from recognised experts in the field in which they want to make improvements. Experience with the 'Breakthrough Series' in the US has shown a variety of achievements including reductions in hospitalisations [16,17].

### The ABCD Project

Building on experience of the CCTs, we carried out the Audit and Best Practice for Chronic Disease (ABCD) project, aiming to examine the impact of improvements in organisational systems on the quality of clinical care in the prevention and management of chronic disease [18,19]. The ABCD project commenced in 2002 and was based on a CQI and action research approach. It was designed with three cycles of assessment, feedback, action planning and implementation of system changes in 12 community health centres in the Top End of Australia's Northern Territory. The ABCD project finished in 2006 with the completion of the third annual CQI cycle. The ABCD project has [18,19]:

▪ achieved a high level of enthusiasm for participation by services, a willingness to engage in, and wide acceptance of the audit, feedback, planning and implementation cycle of quality improvement;

▪ achieved improvements in all key aspects of systems to support chronic illness care;

▪ demonstrated improvements in a number of key indicators of the quality of chronic illness care; and

▪ demonstrated an effective partnership between the research, health policy and health services sectors to enable new ways for working together while generating visible results on the ground.

### The Aim and objectives

The research aim of the Audit and Best Practice for Chronic Disease Extension (ABCDE) project is to inform the operational and policy requirements of a broad-based continuous improvement program in a variety of Indigenous primary health care service settings through an action research process that is designed to enhance the quality of services for the prevention and management of chronic disease. The research will contribute to the evidence base on the impact of collaborative continuous improvement approaches on population health outcomes.

The project objectives are to:

1. Support the implementation of CQI audit tools that generate information for health services to engage in CQI activities;

2. Increase the capacity of Indigenous primary health care organisations to incorporate evidence into practice in order for CQI activities to be 'institutionalised' and sustained;

3. Facilitate development of more effective primary health care policy;

4. Increase the delivery of evidence-based services for the prevention and management of chronic conditions in participating community health centres;

5. Through the above four objectives, to improve chronic disease health outcomes by reducing chronic disease incidence, severity, complications and mortality.

## Methods/design

We will work with a variety of health services/health centres with responsibility for primary health care in Indigenous communities to develop and support a range of tools and strategies relevant to continuous improvement in chronic illness care. The overall approach is designed to meet the collective needs of participating service providers with sufficient flexibility to meet the needs of individual health centres.

### Recruitment of participating health services/centres

A number of services have already agreed to participate or expressed interest in participating in the proposed project. We expect to recruit 40–50 health services from Central Australia and the Top End of the Northern Territory, South Australia, Western Australia and Queensland, and to include both State/Territory funded services and independent Aboriginal Medical Services. A formal agreement to participate will be negotiated with each health centre/service. This agreement will take the form of an exchange of letters that specifies the expectations and requirements of the participating service organisation and the expectations and requirements of the Cooperative Research Centre for Aboriginal Health as the peak research organisation that funded the ABCDE project.

### Continuous Improvement/Action Research Process

Each participating centre will be expected to participate in at least three full annual cycles of audit, systems assessment, feedback, planning and implementation. At least three full cycles will be required to assess sustainability and impact of the quality improvement systems. The project is being implemented over a five year period with timelines for participation tailored to the circumstances of participating health centres.

A staff member from each participating health centre or an organisation with regional responsibility will be designated as responsible for running CQI cycles in each health centre, and will undertake the systems assessment and clinical audit, with training and support provided by research project staff.

Data from the systems assessment and clinical audits will be analysed by research staff. Feedback of results to health centre staff will be conducted in a workshop, with interpretation of the findings being done primarily by health centre staff. The intention of the workshop is to encourage the health centre staff to examine current systems and patterns of clinical care against best-practice guidelines, define priorities for development over the next year and document goals and strategies relevant to their development priorities. We will encourage health service staff to play an active role in facilitation of the workshop. This approach is to encourage health services and government agencies to build capacity in conducting CQI processes at a health service and health centre level and to explore options for how CQI processes may be institutionalised into routine service delivery.

We will explore the potential of using 'collaboratives' to facilitate the sharing of lessons between participating services. The will include the possibility of bringing together representatives of participating health centres with people with expert knowledge and those with strong implementation skills as a means for health service and project staff to draw on the ideas presented to develop strategies for improvement.

This quality improvement process will be based on action learning principles with the aim of encouraging shared learning at various levels of the organisation; greater self-awareness and self-confidence (resulting from new insights and feedback); ability to ask better questions and be more reflective; and improved communications and teamwork [20]. The action research aspect of the process is reflected in the research process being responsive to the needs of participants, feedback received and the findings, with ongoing analysis of findings, reporting back, reflection and refinement of the methodology.

### Continuous Improvement Tools

Each participating centre will use at least three standard tools. All centres will participate in the systems assessment and will use the preventive services and diabetes audit tools. These three tools have been validated and used over successive CQI cycles in a number of health centres, and will provide a set of standard measures that will be available across all participating services. In addition to these tools, participating services may choose to use any of a set of additional tools that will be made available through the project. The project team will provide training and technical support in the use of selected tools and will analyse and report on the data as part of the reporting process described above.

#### 1. Systems assessment

The McColl Institute in the USA has developed a tool for assessment of organisational systems relevant to chronic illness care: the Assessment of Chronic Illness Care (ACIC) scale based on the Chronic Care Model (CCM) developed by the same group [20]. This model of chronic illness care defines the important components of organisational systems [21,22]. The ACIC scale has been used widely in the USA and we have used it successfully with minor adaptations in the ABCD study.

The Chronic Care Model has been the subject of wide international attention and has formed the basis of a World Health Organisation Framework for Innovative Care of Chronic Conditions (ICCC Framework) [1]. In addition to the core components of the CCM, this framework includes attention to the broader policy environment: legislation, financial arrangements for service provision, governance, relationships and collaborations, governmental support, workforce education, etc.

#### 2. Clinical audit tools

The audit tools used in the ABCD project provide for 1) an audit of the delivery of clinical preventive services to the generally well population and 2) an audit of delivery of clinical diabetes services to people known to have diabetes. These tools have been developed and refined based on the experience of the NT Aboriginal Coordinated Care Trials (CCTs) [13,15].

As part of the project these tools are being expanded to audit delivery of clinical services to people known to have 1) hypertension, 2) coronary heart disease, 3) mental health conditions (focusing specifically on schizophrenia and drug induced psychosis as the two most common forms of psychosis in NT Aboriginal communities), and 4) renal disease. Audit tools for hypertension and renal disease were developed for the CCTs and will be refined for use in the proposed project.

Concerns have been expressed about the capacity of smaller health centres to respond to what might be perceived as expanding expectations on services and clinicians imposed by the generation of clinical practice guidelines. Some services may be interested in participating in the project in some way but feel their capacity to participate is limited. Depending on interest we may develop a set of abbreviated audit tools to encourage the participation in the project of smaller centres.

#### 3. Complementary Community Services/Health Promotion audit tool

The delivery of 'Complementary Services' is an important component of the WHO ICCC Framework. The NT Department of Health and Community Services (DHCS) has conducted a Territory-wide audit of health promotion interventions, and reviews of the evidence base to determine best practice health promotion interventions, with a particular emphasis on Indigenous communities. DHCS commissioned the CRCAH to conduct a selected review of Indigenous mental health promotion interventions [23] and a 'review of reviews' for effective interventions in the prevention of chronic disease [24]. On the basis of strong interest from participating health services, we are exploring the possibility of using the evidence generated from this process to develop an appropriate tool for community-based health promotion services that complement the goals and activities of the health centre/service.

### Sampling for clinical audit

In the Coordinated Care Trial Evaluations and in the ABCD project we have successfully used random samples (n = 30) of the clinical records of the generally well adult population (well sample) and of people known to have diabetes (diabetes sample). For the diabetes audit sampling is only relevant in communities with 30 or more people who are known to have diabetes. In communities with smaller numbers the clinical records of all people known to have diabetes are audited. We have established detailed protocols for the sampling process that have been tested and refined in the ABCD project. The sample size has been sufficient to show meaningful trends over time and differences between communities.

With 40 to 50 communities from 4 State/Territories expected to be recruited, the study will not only be able to assess the amount of change over time, but also the variation between communities and characteristics of communities where variation is greatest.

### Process and outcome measures

The project will collect a range of process and outcome data that will be used to examine the research questions relating to effectiveness. These include the identification of factors relating to the capacity of services to take up and sustain ABCD tools and processes, the project impact on service delivery and on outcomes for clients.

In relation to uptake and sustainability of ABCD, aspects of the policy and service environments will be examined. Data will be collected through document analysis and interviews with key informants using an interview framework based on the domains outlined in the WHO ICCC Framework and the Greenhalgh framework on diffusion of innovation.

Process data describing variation in service uptake of ABCD tools and processes will include:

▪ Uptake of the range of audit tools available to participating health centres;

▪ Extent to which use of selected tools is sustained across the full three cycles of the project;

▪ Completeness of data provided by community health centre staff to project staff for processing and feedback;

▪ Level/frequency of support required by health centres/services to effectively participate in the CI processes

▪ Participation in "collaboratives"

▪ Integration of CQI processes into routine service delivery activities. More specifically we will be seeking evidence of chronic illness care relevant CQI processes being written into and implemented through business plans, staff training programs, job descriptions and work practices.

The policy environment will be examined at various levels, including the health service, regional, State/Territory and national levels. Quantitative analysis of data from the adapted ACIC scale will provide a summary measurement of systems and the policy environment. There will also be additional data collected on the detail of the operational systems and policy environment. These data will be available for more specific and in-depth analyses of the characteristics that may be relevant to the capacity of health services to take up, integrate and sustain continuous improvement approaches. Examples of these characteristics include:

▪ At the service level: governance type, medical leadership, staffing levels, staffing profiles, training, clarity of staff roles and responsibilities, stability of staff, employment of population health personnel, availability and use of electronic information systems, availability and use of clinical guidelines, organisation of client care (special clinic, appointments, focus of client care during visit), the role of service initiated and supported self management initiatives; level of regional clinical support eg visiting workers with specific clinical focus;

▪ At the system level: national/State/Territory funding program; national/jurisdictional/regional strategy development, overall budget.

Similarly the interviews that are used to complete the ACIC scale will be used to explore barriers/enhancers to "full" participation in the CQI process.

Outcome measures will be based on data generated by use of the audit tools and will be defined in terms of:

1. *Health outcomes: *hospitalisation and re-hospitalisation within six months, occurrence of complications, and deterioration in function (e.g. exercise capacity, ability to work, ACR). Hospitalisations and re-hospitalisations are generally well documented in clinical records and will be the primary measure of health outcomes. Recording of the development of complications and functional level is more variable and we will examine the potential to use these as outcome measures.

2. *Intermediate health outcomes *(e.g. control of HbA1c, control of blood pressure) The relationship of intermediate health outcomes (such as HbA1c control and BP control) to more definitive outcomes (such as the development of complications) has been well demonstrated. Data on these outcomes are well documented in clinical records and we have used these data in a number of studies to date[13,15,19,25].

3. *Clinical service performance *(proportion of clients for whom services specified in the clinical practice guidelines are delivered, mean proportion of guideline specified services delivered for all clients). The audits of clinical service performance focus on services for which there is the most substantial evidence base for effectiveness. On the strength of the evidence base behind the clinical guidelines the effective delivery of these services is expected to impact on health outcomes. We have used audits of delivery of these services in a number of studies to date[13,15,19,25].

4. *Improvements in the quality of organisational systems *as reflected by the systems assessment. The ACIC scale has been used extensively in the US and we have used an adapted version in the ABCD project to measure improvements in the quality of organisational systems. Analysis of data from the ABCD study indicates that the scale is sensitive to change in organisational systems in our work environment.

5. *Availability of core complementary community based health promotion services*. The assessment of the availability of complementary community-based health promotion services is an area for development for this project. The availability of complementary services is identified as an important domain within the WHO ICCC Framework and is an area of particular interest for DHCS. The DHCS/CRCAH review of the evidence base for health promotion relevant to Indigenous communities will be an important resource for defining 'core' complementary services.

6. *Sustainability*. The measures of integration described above under process measures will be used to assess sustainability of work processes. Sustainability of outcomes will be based on the analysis of trends across the study period and the extent to which the processes supporting these trends are integrated into business plans and work practices.

### Statistical analysis

Community reports will contain results from simple descriptive analyses. The overall project dataset will have a hierarchical structure with individual client data clustered within community health centres, which in turn will be clustered within region and/or governance structures. A multilevel modelling package [26,27]. will be used to examine the relative importance of community level and regional level factors that reflect organisational systems and the broader policy environment that may be important in influencing service delivery and health outcomes. Of particular interest will be improvements in these measures over time as well as differences in the variance of these changes between the various clusters at each level.

### Ethics approval

Participation in this project will require the sharing of de-identified client level data by all participating health services. We understand the sensitivities around the collection and use of such data and have worked through privacy and confidentiality protocols to ensure that high ethical standards are maintained. The research process we are proposing for transmitting and analysing de-identified data will be based on those used in the ABCD project. We have obtained the ethics approval from the Human Research Ethics Committee of NT Department of Health and Community Services and Menzies School of Health Research (including its Indigenous Health Research Ethics Committee) (Reference number 05/10). Project investigators responsible for research oversight of regional hubs will have a key role in ensuring the project meets local ethics committee requirements.

## Conclusion

The ABCDE project will contribute directly to the evidence base on effectiveness of collaborative CQI approaches on prevention and management of chronic disease in Australia's Indigenous communities, and to inform the operational and policy environments that are required to incorporate CQI activities into routine practice.

## Competing interests

The authors declare that they have no competing interests.

## Authors' contributions

RB played a lead role in conceptualisation of study design, development of measurement tools and obtaining research funding. DS played a key role in literature review, development of study design and drafting the manuscript. CC, TW, LC, ST and AB participated in development of study design and facilitated engagement of health services. MD, LO, JC, NC, TN and KG participated in study design and development of measurement tools. All authors were involved in revising the manuscript for important intellectual content and read and approved the final manuscript.

## Pre-publication history

The pre-publication history for this paper can be accessed here:


